# From leptospirosis-induced AKI to AKI at large: the ongoing search for biomarkers to guide RRT initiation

**DOI:** 10.1186/s13613-025-01549-6

**Published:** 2025-09-02

**Authors:** Marie Julien, Yannis Lombardi, Julien Jabot, Cédric Rafat

**Affiliations:** 1 Nephrology Unit, Centre Hospitalier Universitaire Felix Guyon, Saint-Denis, La Réunion, France; 2 Renal Intensive Care Unit, Tenon Hospital, Assistance Publique-Hôpitaux de Paris, Paris, France; 3 Sorbonne Center for Artificial Intelligence (SCAI), Sorbonne University, Paris, France; 4 Sentinelles Network, Pierre Louis Institute for Epidemiology and Public Health, INSERM and Sorbonne University, Paris, France; 5 Intensive Care Unit, Centre Hospitalier Universitaire, Saint-Denis, La Réunion, France

Dear Editor-in-chief

We thank Dr. Wu and Dr. Huang for their insightful comments. We fully concur with their key message: plasma creatinine is an unreliable marker for defining the timing of renal replacement therapy (RRT) initiation.

In fact, we wish to take the argument one step further and question whether any variable currently used to assess the severity of Acute Kidney Injury (AKI)—whether alone or in combination—can reliably serve as a trigger for RRT initiation. The core trade-off in the AKI staging system is between the pathophysiological complexity of the condition and the simplicity required for prognostication. Although it may seem intuitive to equate severity with the need for RRT, doing so overlooks the primary purpose of RRT: the restoration of physiological homeostasis. Furthermore, it remains unclear whether a handful of broad and imperfect physiological indicators—including as blood urea nitrogen—can adequately reflect the diverse pathophysiological landscape of AKI in the ICU.

Returning to leptospirosis-induced AKI (AKI-L), its added complexity lies in a multidimensional pathophysiology that extends beyond the archetypal sepsis-associated acute tubular necrosis, which largely underpinned the AKIKI trial [1] and subsequent studies [2]. Hypovolemia and sodium wasting are salient features of AKI-L, supporting a conservative management approach, whereas tubulointerstitial injury suggests ongoing parenchymal kidney damage, potentially warranting RRT.

These same considerations are relevant when evaluating the risk of chronic kidney disease (CKD). If patients with AKI-L who receive RRT are indeed at higher risk of progressing to CKD [3], consistent with the authors’ interpretation and the hypothesis of an iatrogenic Hemodynamically Instable Renal Replacement Therapy (“HIRRT”) phenomenon, the overarching determinant still appears to be the severity of AKI per se, regardless of the utilization of RRT. Accordingly, progression to kidney fibrosis and thus CKD has been connected to tubulointerstitial disease [4].

Until these issues are clarified, clinicians should adhere to the framework established by AKIKI and subsequent trials. This guidance remains applicable to patients with AKI-L [5].

## Data Availability

Not applicable.

## Abbreviations

AKI: Acute Kidney Injury
AKI-L: Leptospirosis-induced AKI
CKD: Chronic kidney disease
RRT: Renal replacement therapy
